# Incidence, survival time and associated factors of virological failure among adult HIV/AIDS patients on first line antiretroviral therapy in St. Paul’s Hospital Millennium Medical College—A retrospective cohort study

**DOI:** 10.1371/journal.pone.0275204

**Published:** 2022-10-13

**Authors:** Demeke Eshetu Andarge, Haimanot Ewnetu Hailu, Takele Menna

**Affiliations:** Department of Public Health, Saint Paul’s Hospital Millennium Medical College, Addis Ababa, Ethiopia; Public Library of Science, UNITED KINGDOM

## Abstract

**Introduction:**

Human Immune deficiency Virus or Acquired Immune deficiency Syndrome (HIV/AIDS) is a pandemic affecting millions around the world. The 2020 the Joint United Nations Programme on HIV/AIDS report stated that the estimated number of people living with HIV (PLHIV) is 38 million globally by 2019. Ethiopia is among HIV high burden countries in Africa. By 2021, PLHIV in Ethiopia is estimated to be 754, 256. Globally out of 25.4 million PLHIV on ART, 41% reported virally non-suppressed. According to UNAIDS, the estimated viral non-suppression in Ethiopia is about 27%.

**Methodology:**

A hospital based retrospective cohort study was conducted among 323 patients who were enrolled to ART from July 2016 to December 2020. The medical records of study participants were selected using simple random sampling technique & data was collected using data extraction checklist. The collected data was entered and cleaned using SPSS V.25. Kaplan–Meier is used to estimate the cumulative hazard of virological failure at different time points. During bivariate analysis variables with p<0.25 were taken for Multivariate Cox regression analysis to assess predictors of virological failure & statistically significant association was declared at p<0.05 with 95% confidence interval.

**Result:**

The overall incidence rate of virological failure was 1.75 per 1000 months of observations. The mean survival time of virological failure was 14.80 months. Disclosure of sero-status (AHR = 0.038, 95% CI: 0.008–018), poor adherence (AHR = 4.24, 95% CI: 1.04–16), having OIs (Opportunistic infections) (AHR = 4.59, 95% CI: 1.17–18) and use of cotrimoxazole (CPT) prophylaxis (AHR = 0.13, 95% CI: 0.026–0.68) have shown statistically significant association with virological failure.

**Conclusion:**

The incidence of virological failure among patients on first line ART in St. Paul’s hospital is low. Disclosure of sero-status, poor adherence, having OIs and use of CPT prophylaxis were associated with virological failure. Therefore, a due attention needs to be given to these factors in order to minimize virological failure in patients on ART.

## Introduction

Human Immune deficiency Virus or Acquired Immune Deficiency Syndrome (HIV/AIDS) is a pandemic affecting millions around the world. The 2020 Joint United Nations Programme on HIV/AIDS (UNAIDS) report stated that the estimated number of people living with HIV was 38 million globally by 2019. Despite the efforts to control this pandemic, the infection rate is high; in 2019, the estimated number of new infections was 1.7million. Of 38 million people living with the Human immune deficiency virus, merely 25.4 million are taking antiretroviral therapy while the remaining are waiting. In 2019, HIV/AIDS claimed life of 690,000 people [1]. About a 74.9million people have been infected with HIV since it became a pandemic and 32 million have died of AIDS-related illness [2].

The vast majority of people living with HIV are located in Low- and Middle- Income Countries (LMIC), with an estimated 68% living in sub-Saharan Africa. Among this group, 20.6 million are living in East and Southern Africa where 800,000 new HIV infections were recorded in 2018 [2].

According to an estimate by the ministry of health, the number of PLHIV in Ethiopia by the years 2020 and 2021 will be 745,719 and 754,256 respectively. Antiretroviral drugs are made available in the country for the first time in 2003 and then free in 2005 [3,4]. UNAIDS has reported that only two-thirds of the 690000 people living with HIV in Ethiopia in 2018 are on treatment [5]. Ethiopian Demographic and Health Survey (EDHS) 2016 indicated that the national prevalence of HIV in Ethiopia was 0.96% [6]. The report from Ethiopia Population-based HIV Impact Assessment (EPHIA) indicates that the 2017–2018 prevalence of HIV among adults aged 15–64 years in urban Ethiopia was 3.0%. In Ethiopia, 81% of all people living with HIV are on treatment and 73% of them were virally suppressed which makes the 27% non-suppressed [7].

The prevalence of HIV/AIDS in Addis Ababa, according to EPHIA, 2017–2018 was 3.1% [7]. The ministry of health estimated people living with HIV in Addis Ababa by the years 2020 and 2021 will be 132,524 and 133,720 respectively [3,4]. The HIV care and treatment service coverage in Addis Ababa indicated 74.6% and viral load testing coverage is about 60% with 87.5% viral suppression among those who received viral load testing [8].

HIV has a lifelong treatment, which is monitored by various means. Viral load (VL) testing is among the mechanisms, which gain wider acceptance these days. Measuring VL can help to distinguish between treatment failure and non-adherence. Studies in 2013 WHO recommended viral load testing [9]. WHO defined virological failure (VF) as, plasma viral load above 1000 copies/ ml based on two consecutive viral load measurements after 3 months, with adherence support [10].

As VL testing is becoming routine across countries, measuring its impact and progress towards achieving the UNAIDS target that 90% of people receiving antiretroviral therapy have suppressed viral loads by 2020 (as part of the 90–90–90 targets) is very important [11].

Viral load suppression can be a performance indicator for ART programs. Regular VL-monitoring allows identification of suboptimal adherence. This recommendation is based on research demonstrating that viral suppression is associated with decreased HIV disease progression and mortality among people living with the human immune virus (PLHIV), and the prevention of HIV transmission to sexual partners [12,13].

Virological status follow-up will give initial and precise information on the possibility of treatment failure, the necessity to change regimens, lessen mutations that result from drug resistance, and bring desired outcomes. Therefore, VL tests save patients from being needlessly switched to medicines that are more expensive or left to continue on ineffective therapy that can lead to drug resistance and ultimately death [14,15].

The problem has multidimensional consequences on the individual, family, community, economy, and the health system at large. Virological failure is not a sole entity; it goes hand in hand with drug resistance. VF already endangered the handful of drugs that are in use in a fight against the virus. Even though maintaining a low viral load is important for patients to prevent the progression of AIDS and associated co-infections and the rate of HIV infection in Addis Ababa is high, the evidence on virological failure, survival time and the associated factors are limited. Therefore, this study is aimed to fill this gap in producing evidence that can be useful in making an informed decision. In addition, it will contribute to the realization of the 90-90-90 treatment target and achieve sustainable development goal 3.

## Method and materials

A retrospective cohort study was conducted on HIV infected participants on a WHO recommended antiretroviral therapy (ART) and enrolled in Saint Paul’s Hospital Millennium medical college between July 2016 and December 2020.

St. Paul’s Hospital Millennium Medical College is located in Addis Ababa, the capital. The ART service was started in 2003 and currently more than 5080 clients are getting the service free of charge.

Sample size was determined using Epi Info™ Version: 7.2.1.0 StatCalc by considering 95% confidence level, 80% power, unexposed to exposed group ratio of one and taking the key predictor of VF (BMI <16 which gave the largest sample size among the variables) from a previous study in Woldia and Dessie hospitals and Waghimra zone. Therefore, the calculated minimum sample size is 308 and by considering a 10% non-response rate the final sample is 340.

The outcome variable was virological failure is plasma viral load above 1000 copies/ ml based on two consecutive viral load measurements after 3 months, with adherence support. The potential associated factors included age, gender, education, marital status, disclosure status, occupational status, BMI, base line drug regimen, cotrimoxazole prophylaxis, base line functional status, WHO stage, adherence to treatment, TB/HIV co-infection, opportunistic infections other than TB and CD4 cell count. A simple random sampling technique was used to select participants who are greater than 15 years of age and on ART at least for 10 months before data collection. Data was extracted from patient cards using a structured checklist prepared in English adapted from Ethiopian Federal Ministry of Health ART clinic intake and follow up form.

Inclusion and exclusion criteria: The inclusion criteria include: clients aged 15years or older, who were on treatment for at least ten months and on first line ART. Transfer in and those without viral load test were excluded from the study.

Operational definition: Censored are those patients complete the follow up, transferred out or lost without developing virological failure. Adherence is defined as follows: Clients on ART with >95% adherence are considered to have good adherence, those with 85–94% adherence are fair and those with <85% are considered to have poor adherence [16]. History of Opportunistic infection or Tuberculosis indicates whether the patient has history of any Opportunistic infection including Tuberculosis. On the other hand, recent infection is whether the person currently has TB or any Opportunistic infection.

Data were entered, cleared and analyzed using SPSS version 25. Descriptive statistics was used to describe demographic, clinical and medication-related characteristics of patients. The Kaplan-Meier method was used to estimate the cumulative incidence of virological failure at different time points. Incidence of virological failure was calculated using Person months (PM) observation. Cox proportional hazards model was used to identify factors significantly associated with virological failure and to control confounding factors. To control the confounders, a multivariable model was developed for a priori confounders including age and sex which are selected based on existing literature. A p- value of less than 0.05 with 95% was used to declare statistical significance.

This study was approved by the SPHMMC Institutional review Board. An official letter of permission was obtained from the Hospital to access the data from the record of patients that is fully anonymized before we accessed them. An informed verbal consent was obtained from the study participants. The obtained information was kept confidential and only be used for research purpose.

## Result

### Socio-demographic characteristics

From July 2016 to December 2020, 640 adult HIV patients on first-line ART were enrolled in St. Paul’s hospital millennium medical college ART clinic and 340 medical record cards were selected using simple random sampling of which 17 medical record cards were excluded due to missed charts and incomplete data. As a result, a total of 323 patient cards were included in the analysis

The mean age of the patients was 36.86 (SD±9.8) with minimum age of 16 and maximum 70 years. More than half, 188 (58.2%) of the patients were female. One hundred seven (33.1%) attended primary level of education and 123(38.1%) were with secondary education. Half of the participant in this study 163(50.5%) were married and 142(43.7%) were employed. More than 3/4^th^ of the participants disclosed their HIV status at least for one person (Table 1).

**Table 1:** Base line Socio-demographic characteristics of first line ART clients in St. Paul’s hospital millennium medical college in Addis Ababa, Ethiopia from July 2016 to December 2020 (N = 323).

Variable	Frequency (%)
Age group	
under 20	9(2.8)
21–30	92(28.5)
31–40	121(37.5)
41–50	74(22.9)
51 and above	27(8.3)
Gender	
female	188(58.2)
male	135(41.8)
Education	
No formal education	37(11.5)
Primary	107(33.1)
Secondary	123(38.1)
College and above	56(17.3)
Marital status	
Married	163(50.5)
Never married	142(44)
Divorced/widowed	18(5.6)
Disclosure status	
Disclosed	252(78)
Not disclosed	57(17.6)
unknown	14(4.3)
Occupational status	
employed	141(43.7)
self employed	42(13.0)
unemployed	132(40.9)
Others	8(2.5)

### Baseline clinical and anti-retroviral medication-related characteristics

Majority of the participants 202(62.5%) had normal body mass index. Two hundred sixty nine, 269(83.3%) of the total participants were on Efavernez (EFV) based first line ART drug regimen. More than half of the patients, 174(53.9%) took Cotrimoxazole preventive therapy (CPT). Nearly all 320(99.1%) patients in the study could perform their routine activities. More than half of the patients 193(59.8%) had baseline WHO clinical stage I/II and 247(76.5%) had good ART adherence status. On the other hand, 46(14.2) had TB/HIV co-infection and 82(25.4%) experienced OI other than TB. More than one third (36.5%) had a base line CD4 count of less than 200 copies ml and the mean month of developing virological failure was 30(SD±12) with minimum 10.4 and maximum 53.26 months respectively (Table 2).

**Table 2:** Baseline clinical and antiretroviral medication-related information among adult HIV patients on first-line ART in St. Paul’s hospital millennium medical college in Addis Ababa, Ethiopia from July 2016 to December 2020 (N = 323).

Variable	Frequency (%)
BMI category (Kg/m^2^)	
under18.5	44(13.6)
18.5–24.9	202(62.5)
25–29.9	65(20.1)
30 and above	12(3.7)
Base line drug regimen	
Nevirapine based	11(3.4)
Efavirenz based	269(83.3)
DTG based	43(13.3)
Cotrimoxazole prophylaxis*	
yes	174(53.9)
no	149(46.1)
Base line functional status	
Working	320(99.1)
Ambulatory	3(0.9)
WHO stage	
I/II	193(59.8)
III	79(24.5)
IV	51(15.8)
Adherence to treatment	
Fair/poor	76(23.5)
Good	247(76.5)
TB/HIV co-infection	
yes	46(14.2)
no	277(85.8)
OIs other than TB	
yes	82(25.4)
no	241(74.6)
CD4 (cells/mm^3^)	
200 and below	118(36.5)
201–350	75(23.2)
351–500	52(16.1)
501 and above	60(19.7)
Missing**	18(5.6)

*taken cotrimoxazole prophylaxes at any time in the follow up time.

### The incidence of virological failure

All the participants of the study were followed for different periods with a total of 9,698.36 person-months (PM) of observations. The first patient developed the event (VF) after 10.4 months of follow up and the last after 53.3 months. Seventeen, 17(5.3%) of patients developed VF during the follow-up period. The overall incidence rate of VF in this follows up was 1.75 events per 1000 PM of observations. The cumulative hazard of VF at 12, 24, 36 and 48 months were 1.2%, 3.7%, 5.5% and 7.6% respectively.

**Fig 1:**
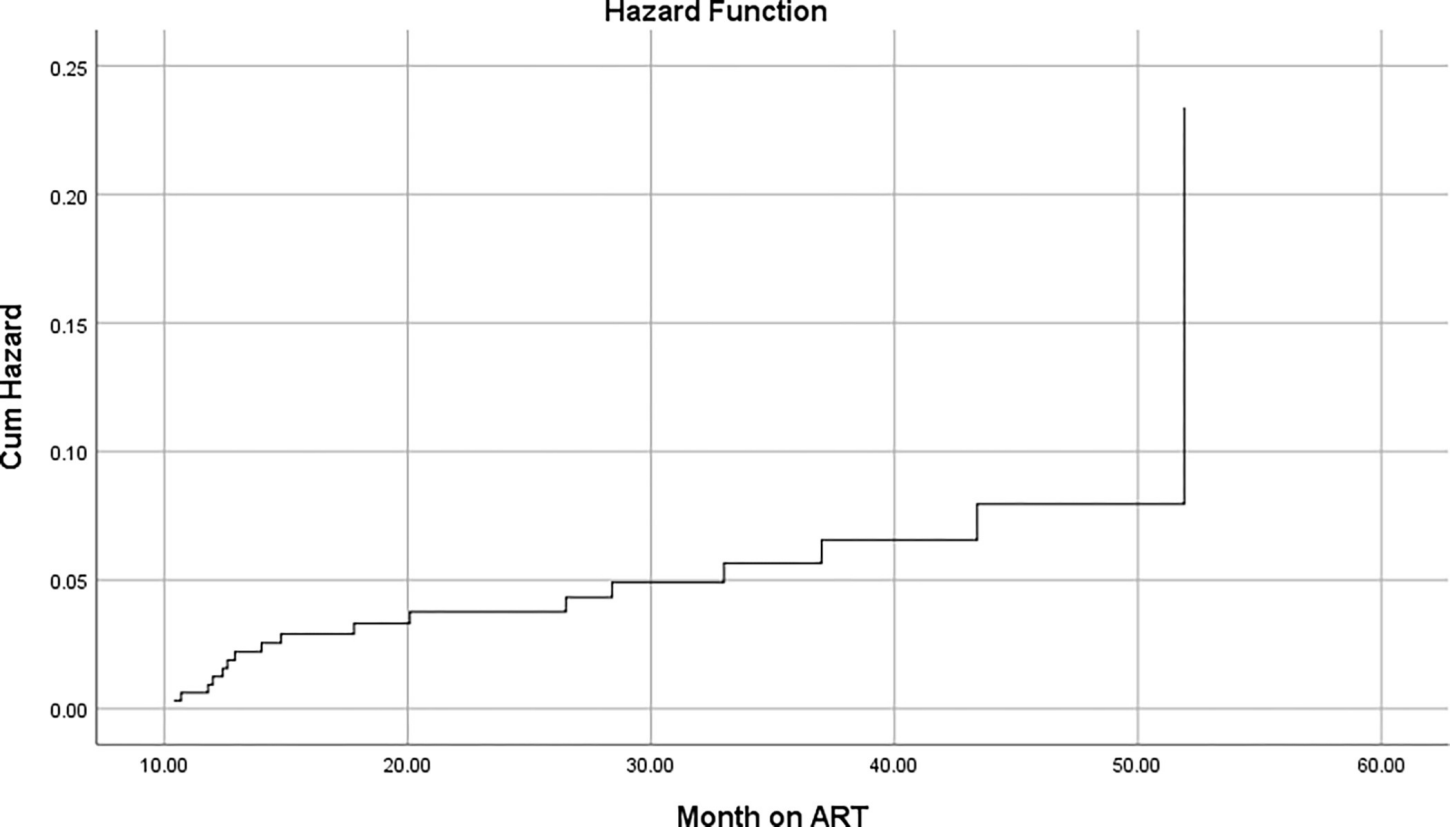
Cumulative incidence of VF among first line adult ART patients in St. Paul’s hospital millennium medical college from July 2016 to December 2020.

A graph of the Kaplan Meier (KM) failure function was used to describe the cumulative IR of virological failure over the follow-up period (Fig 1).

### Survival time of virological failure

**Fig 2:**
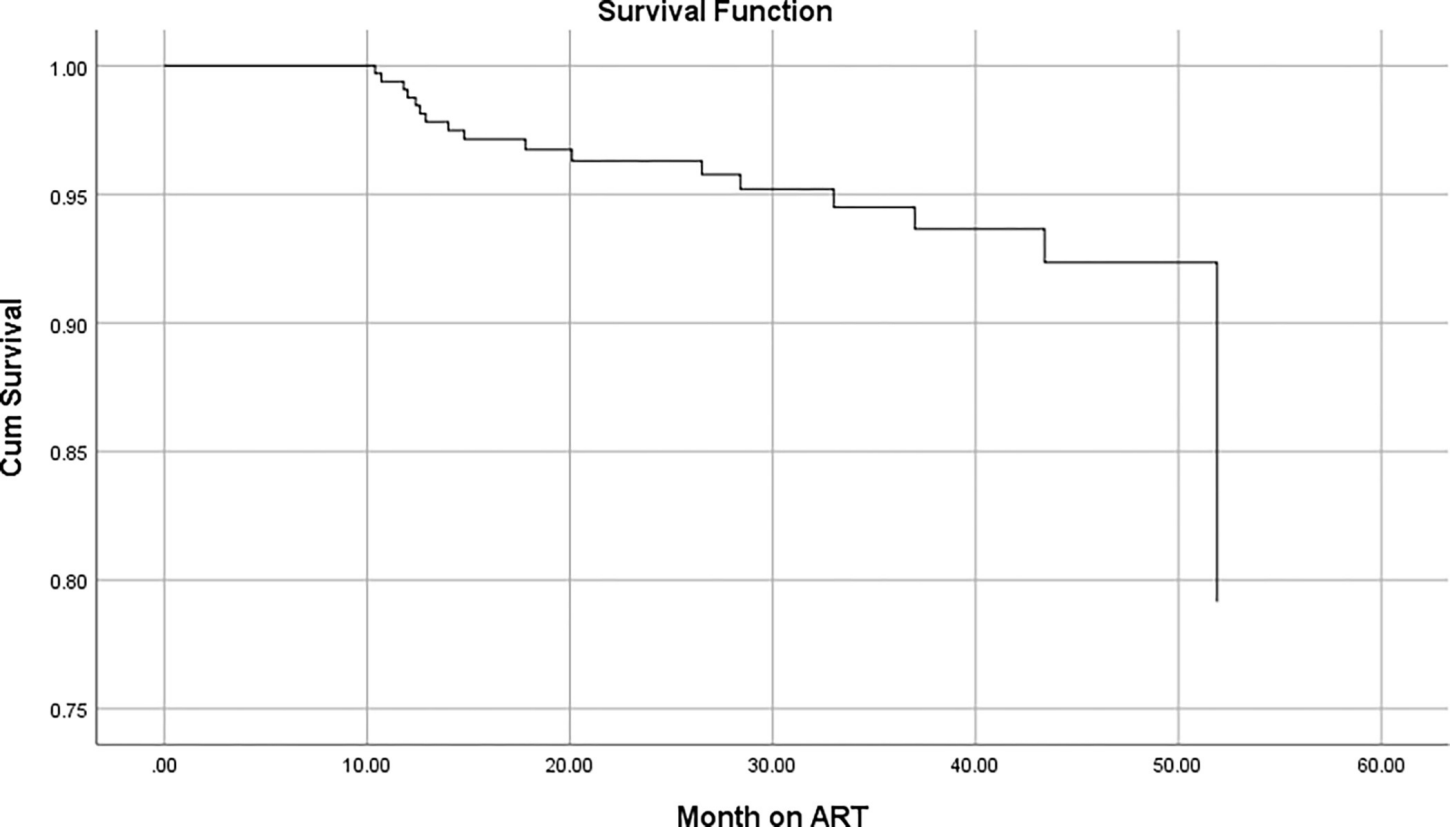
Kaplan-Meier’s survival graph of patients on ART in St. Paul’s hospital millennium medical college from July 2016 to December 2020.

The cumulative probability of surviving or being free from the event of interest, VF at the end of 12, 24, 36 and 48 months was 98.76%, 96.30%, 94.49% and 92.35% respectively (Fig 2). The mean survival time of virological failure was 14.8 months.

### Factors associated with virological failure

The risk of developing VF among participants disclosed their HIV status decreased by 96.2% compared to their counterparts (AHR) (AHR = 0.038, 95% CI: 0.008–0.18). Patients with poor treatment adherence were four times more likely to develop VF compared to those with good adherence (AHR = 4.24, 95% CI: 1.04–16). Similarly, patients having history of OIs were at four-fold risk of VF compared to those without OIs (AHR = 4.59, 95% CI: 1.17–18). In addition, patients taking cotrimoxazole prophylaxis are 87% less likely to develop to VF compared to those who are not on the prophylaxis (AHR = 0.13, 95% CI: 0.026–0.68) (Table 3).

**Table 3:** Factors associated with virological failure among first ART clients in St. Paul’s Hospital Millennium Medical College, Addis Ababa, Ethiopia from July 2016 to December 2020.

Variable	Event	Censored	P-value	CHR 95% CI	P-value		AHR 95% CI
Gender							
Male	12	123	**0.023**	3.35(1.18–9.51)	0.73		1.26(0.35–4.59)
Female	5	283		1			1.000
Education status							
No formal education	2	35	0.31	0.44(0.09–2.2)	0.79		1.38(0.12–16.55)
Primary	3	104	**0.04**	0.22(0.05–0.91)	0.16		0.19(0.019–1.89)
Secondary	6	117	0.09	0.37(0.12–1.2)	0.52		0.56(0.09–3.22)
college & above	6	50		1			1.000
Base line functional status							
Working	16	304	**0.069**	0.15(0.020–1.16	0.96		0.92(0.039–21)
Ambulatory	1	2		1			1.000
Marital status							
Married	7	156	**0.21**	0.36(0.07–1.74)	0.75		1.52(0.12–19.6)
Never married	8	134	0.36	0.48(0.1–2.3)	0.68		1.75(0.13–23.85)
Divorced/widowed	2	16		1			1.000
Disclosure status							
Disclosed	6	246	**0.000**	0.11(0.04–0.29)	**0.000**		0.038(0.008–0.18)
Not disclosed	11	46		1			1
Adherence to treatment							
Fair/poor	11	65	**0.00**	6.3(2.31–17.09)		**0.040**	4.24(1.06–16.9)
Good	6	241		1			1
WHO stage of participant							
WHO stage I/II	2	191	**0.000**	0.041(0.009–0.185)		0.27	0.29(0.03–2.62)
WHO stage III	3	76	0.004	0.15(0.043–0.55)		0.18	0.29(0.05–1.77)
WHO stage IV	12	39		1			1.000
TB/HIV co-infection							
Yes	9	37	**0.000**	7.23(2.77–18.8)		0.11	3.07(0.76–12.36
No	8	269		1			1.000
OIs other than TB	8	269		1			1.000
Yes	12	70	**0.000**	6.97(2.45–19.7)		** 0.029**	4.59(1.17–18.07)
No	5	236		1			1.000
Cotrimoxazole prophylaxes (CPT)							
Yes	11	163	0.18	0.14(0.53–3.9)		**0.015**	0.13(0.026–0.68)
No	6	143		1			1
CD4 count							
CD4 = <200	14	104	**0.051**	4.45(0.99–19.9)		0.216	3.56(0.476–26.66)
CD4 201–350	1	74	0.490	0.44(0.040–4.8)		0.88	0.82(0.062–10.82)
CD4 351–500	0	52	0.970	0		0.919	0.000
CD4 > = 501	2	58		1			1.000

## Discussion

This study assessed the incidence rate of VF and associated factors among adult HIV/AIDS patients on first- line ART attending St. Paul’s hospital millennium medical college in Addis Ababa, Ethiopia.

The overall incidence rate of VF in this study was 1.75 per 1000 PM of observation (95 CI: 0.024–0.068). This result was lower than a finding of similar study conducted in Amhara referral hospital, which was 4.9 per 1000 PM [16]. The reason for discrepancy may be the high proportion of underweight patients in the Amhara hospital study as compared to the current study that is 32.24% vs 13.6%. Studies illustrated that underweight clients on ART are at more risk of VF [17]. In addition, the study in Amhara region hospital reported that proportion of patients with no formal education and primary education was 51% higher compared to the current study, which was 44%, and studies suggested that patients with lower educational level are at higher risk of developing VF [18]. The socio-cultural difference between the study settings may be another contributing factor for the difference in the findings. Majority of the participants of this study were dwellers of the capital, Addis Ababa, with better access to service and information, which might have a contribution for lower Virological Failure.

A similar study in Adama reported the incidence rate of 2.1 per 1000 PM, which was more or less comparable with the result of current study [19]. This could be the physical proximity between the two cities and socio-cultural similarities.

A study in South Africa indicated 3.8 events per 1000 PM observation which was higher than this study [20]. The cohort in South Africa took patients on treatment for a relatively longer duration compared to this study. As indicated in some studies the increase in treatment duration increases the risk of developing VF. The possible explanation for this includes tolerance developed by the patients against advices by professionals, pill fatigue and/ or mutation of the virus over time [21,22]. The other possible reason that caused the discrepancy could be the large sample size used in the South African study.

The incidence rate reported from Ugandan study was 4.8 per 1000 PM, which was higher than the finding in our study [23]. The residence area of the study participants could be an explanation for the observed difference. More than three fourth (76.8%) of the participants were from rural areas of Uganda as opposed to the current study of which majority of the patients were from the capital. This could be explained by the fact that urban residents have better awareness of the treatment and the risk for VF is lesser. The other possible reason could be that the age of the study participants; where 44.1% of the Ugandan cohort were the below the age of 30 years while the proportion of the same age group in current study was 31.3%. As different articles suggest the young age group is highly vulnerable to VF [19,24,25].

Another similar study from India reported a VF incidence rate of 8.9 event per 1000 PM observation [26]. The cause for variation of the two results was the study in India excluded those patients with higher CD4>350 and WHO stage II and I. Patients with advanced WHO staging and lower CD4 count are at risk of developing VF [14,27,28].

The finding of the current study exhibited that the mean survival time of virological failure was 14.8 months. This could be due to the fact that, when clients start a treatment there might be issues with acceptance of the treatment and intolerance to adverse effects may develop which this in turn affects adherence and subsequent development of virological failure.

The study revealed that participants who disclosed their HIV status were 96.2% less likely to develop virological failure. The possible explanation for this could be that, ART is a lifelong treatment and care that demand the support of others. Those who disclosed their status could get the necessary moral, psychological and material support when they are in need in contrast to their counterparts. Patients who declared their HIV status to their loved ones and friends can freely take their medication in front of family members, even reminded by family members and friends to take drugs on time. This study finding is in line with studies in Zimbabwe [29] and Uganda [23].

The other factor found to be significantly associated with VF is poor adherence. Patients with poor adherence are more than 4 times at risk of virological failure than those with good adherences. This finding is in agreement with studies in India [26], Addis Ababa [8] Mekele [14], Gondar [21], Dessie and Woldia [27], Adama [19] and Amhara regional hospitals [16]. In fight against infections like HIV, adhering to the treatment is a key, otherwise it creates a situation that leads to development of resistant varieties that cannot be controlled by the medications at hand. Patients with poor adherence are exposed to CD4 cell reduction, which in turn affects their immune status, and rise viral load [20].

Similarly, the hazard of VF of patients with OIs is 4 times as compared to patients without OIs. Some studies indicate that the presence of other infections with HIV could affect the ART care service [30,31]. Unfortunately, OIs are common among HIV patients. Some OIs are recurrent in nature and made the patient take multiple drugs, this in turn made patient to focus on current illness and give less attention to the chronic condition and pill fatigue may occur. When patient overwhelmed by pill burden they fail to take their ART properly leading to VF [32].

Patients on CPT prophylaxis were 86.6% less likely to develop VF compared to those who did not take the prophylaxis. This happens because CPT increases the CD4 cells of patients and improves their immune status. When the immune system of the patients improve the viral multiplication decreases making them at lower risk of VF [16].

Limitations of this study were, use of secondary data, socio-economic factors like income, behavioral factors like alcohol consumption, smoking and drug use and psychological factors like depression were not included. In addition, though multivariable analyses were adjusted for a known confounder, residual confounding factors cannot be ruled out and the results should be interpreted with caution.

## Conclusion

The incidence rate of virological failure was low among HIV patients on the first line ART at St. Paul’s hospital millennium medical college. Higher level of virological failure is observed at early stage of the treatment. The mean survival time of virological failure was 14.8 months. Poor adherence to treatment, failure to disclose HIV status, having OIs and not using cotrimoxazole prophylaxis were the factors associated with increased risk of virological failure. Due attention needs to be given for patients with this conditions during the follow up time.

## Supporting information

S1 Data (SAV)

## References

[1] UNAIDS. UNAIDS DATA 2020. 2020.

[2] UNAIDS. POWER TO THE PEOPLE. 2019.

[3] Federal HIV/AIDS Prevention and Control Office. HIV Epidemic Estimates 2017–2021. 2015.

[4] Ethiopia IIfPHC-. HIV Related Estimates and Projections for Ethiopia–2017. 2017.

[5] UNAIDS. UNAIDS data 2018. 2018.

[6] Central Statistical Agency (CSA) [Ethiopia] and ICF. Ethiopia Demographic and Health Survey 2016. 2016.

[7] Institute EPH. Ethiopia Population-based HIV Impact Assessment (EPHIA) 2017–2018: Final Report. 2020.

[8] Adal M. Systematic review on HIV situation in Addis Ababa, Ethiopia. BMC Public Health. 2019;19(1):1544. doi: 10.1186/s12889-019-7885-8 31752778 PMC6873765

[9] WHO. The use of antiretroviral drugs for treating and preventing HIV infection Recommendations for a public health approach. 2016. 27466667

[10] WHO. WHO definitions of clinical, immunological and virological failure for the decision to switch ART regimens. 2015. 2015.

[11] WHO. Considerations for Developing a Monitoring and Evaluation Framework for Viral Load Testing. 2019.

[12] Nicholas S, Poulet E, Wolters L, Wapling J, Rakesh A, Amoros I, et al. Point-of-care viral load monitoring: outcomes from a decentralized HIV programme in Malawi. J Int AIDS Soc. 2019;22(8):e25387–e. doi: 10.1002/jia2.25387 31441242 PMC6706700

[13] El-Sadr WM, Rabkin M, Nkengasong J, Birx DL. Realizing the potential of routine viral load testing in sub-Saharan Africa. J Int AIDS Soc. 2017;20 Suppl 7(Suppl 7). doi: 10.1002/jia2.25010 29130621 PMC5978658

[14] Hailu GG, Hagos DG, Hagos AK, Wasihun AG, Dejene TA. Virological and immunological failure of HAART and associated risk factors among adults and adolescents in the Tigray region of Northern Ethiopia. PloS one. 2018;13(5):e0196259. doi: 10.1371/journal.pone.0196259 29715323 PMC5929526

[15] UNAIDS. The need for routine viral load testing. 2016.

[16] Agegnehu CD, Merid MW, Yenit MK. Incidence and predictors of virological failure among adult HIV patients on first-line antiretroviral therapy in Amhara regional referral hospitals; Ethiopia: a retrospective follow-up study. BMC Infectious Diseases. 2020;20(1):460. doi: 10.1186/s12879-020-05177-2 32611405 PMC7329399

[17] Emagnu A, Abay Z, Bulti AB, Animut Y. Determinants of Virologic Failure among Adult HIV Patients on First-Line Antiretroviral Therapy at Waghimra Zone, Northern Ethiopia: A Case-Control Study. Advances in Public Health. 2020;2020:1929436.

[18] Ayele G, Tessema B, Amsalu A, Ferede G, Yismaw G. Prevalence and associated factors of treatment failure among HIV/AIDS patients on HAART attending University of Gondar Referral Hospital Northwest Ethiopia. BMC immunology. 2018;19(1):37. doi: 10.1186/s12865-018-0278-4 30558580 PMC6296084

[19] Endebu T, Deksisa A, Moges T, Kisi T, Ensermu T. Incidence of Virological failure and associated factors among adult HIV-positive patients on first line antiretroviral therapy regimen, Central Ethiopia. International Journal of HIV/AIDS Prevention, Education and Behavioural Science. 2019;5:8.

[20] Johnston V, Fielding KL, Charalambous S, Churchyard G, Phillips A, Grant AD. Outcomes following virological failure and predictors of switching to second-line antiretroviral therapy in a South African treatment program. J Acquir Immune Defic Syndr. 2012;61(3):370–80. doi: 10.1097/QAI.0b013e318266ee3f 22820803 PMC3840925

[21] Bayu B, Tariku A, Bulti AB, Habitu YA, Derso T, Teshome DF. Determinants of virological failure among patients on highly active antiretroviral therapy in University of Gondar Referral Hospital, Northwest Ethiopia: a case-control study. HIV/AIDS (Auckland, NZ). 2017;9:153–9. doi: 10.2147/HIV.S139516 28848364 PMC5557910

[22] Asfaw AB, Nigusie A, Shewanow T, Gudina EK, Getnet M, Amdisa D, et al. Determinants of First-line Antiretroviral Treatment Failure Among Patients on Antiretroviral Therapy in Public Hospitals Jimma, Southwest Ethiopia a Case-Control Study. Rehabilitation. 2019;4(2):13–24.

[23] Izudi J, Alioni S, Kerukadho E, Ndungutse D. Virological failure reduced with HIV-serostatus disclosure, extra baseline weight and rising CD4 cells among HIV-positive adults in Northwestern Uganda. BMC Infectious Diseases. 2016;16(1):614. doi: 10.1186/s12879-016-1952-x 27793124 PMC5084428

[24] Carriquiry G, Giganti MJ, Castilho JL, Jayathilake K, Cahn P, Grinsztejn B, et al. Virologic failure and mortality in older ART initiators in a multisite Latin American and Caribbean Cohort. J Int AIDS Soc. 2018;21(3):e25088–e. doi: 10.1002/jia2.25088 29569354 PMC5864576

[25] Namale G, Kamacooko O, Bagiire D, Mayanja Y, Abaasa A, Kilembe W, et al. Sustained virological response and drug resistance among female sex workers living with HIV on antiretroviral therapy in Kampala, Uganda: a cross-sectional study. Sexually transmitted infections. 2019;95(6):405–11. doi: 10.1136/sextrans-2018-053854 31266818 PMC6824617

[26] Shet A, Neogi U, Kumarasamy N, DeCosta A, Shastri S, Rewari BB. Virological efficacy with first-line antiretroviral treatment in India: predictors of viral failure and evidence of viral resuppression. Tropical medicine & international health: TM & IH. 2015;20(11):1462–72. doi: 10.1111/tmi.12563 26146863

[27] Ahmed M, Merga H, Jarso H. Predictors of virological treatment failure among adult HIV patients on first-line antiretroviral therapy in Woldia and Dessie hospitals, Northeast Ethiopia: a case-control study. BMC Infectious Diseases. 2019;19(1):305. doi: 10.1186/s12879-019-3924-4 30943903 PMC6448227

[28] Ayalew MB, Kumilachew D, Belay A, Getu S, Teju D, Endale D, et al. First-line antiretroviral treatment failure and associated factors in HIV patients at the University of Gondar Teaching Hospital, Gondar, Northwest Ethiopia. HIV/AIDS (Auckland, NZ). 2016;8:141–6. doi: 10.2147/HIV.S112048 27621669 PMC5015875

[29] Sithole Z, Mbizvo E, Chonzi P, Mungati M, Juru TP, Shambira G, et al. Virological failure among adolescents on ART, Harare City, 2017- a case-control study. BMC Infectious Diseases. 2018;18(1):469. doi: 10.1186/s12879-018-3372-6 30227831 PMC6145182

[30] Shoko C, Chikobvu D, Bessong PO. A Markov model for the effects of virological failure on HIV/AIDS progression in tuberculosis co-infected patients receiving antiretroviral therapy in a rural clinic in northern South Africa. South African medical journal = Suid-Afrikaanse tydskrif vir geneeskunde. 2020;110(4):313–9. doi: 10.7196/SAMJ.2020.v110i4.13934 32657744

[31] Abebaw A, Taddele M, Alem G, Birlew T. Magnitude of virologic failure and associated factors among adult patients on antiretroviral therapy at Debre Markos Referral Hospital, Northwest Ethiopia. 2018.

[32] Endalamaw A, Mekonnen M, Geremew D, Yehualashet FA, Tesera H, Habtewold TD. HIV/AIDS treatment failure and associated factors in Ethiopia: meta-analysis. BMC Public Health. 2020;20(1):82. doi: 10.1186/s12889-020-8160-8 31959136 PMC6971997

