# Supplementary material for: Incidence, survival time and associated factors of virological failure among adult HIV/AIDS patients on first line antiretroviral therapy in St. Paul’s Hospital Millennium Medical College—A retrospective cohort study
Source: PLoS One. 2022 Oct 13;17(10):e0275204. doi: 10.1371/journal.pone.0275204 (PMC9560068; doi:10.1371/journal.pone.0275204)
Supplement: Attachment — Submitted filename: Response to Reviewers.docx [file pone.0275204.s002.docx]

**Response to reviewers**

We appreciate the reviewers’ comments. The followings are our point-by-point responses:

1. Is the manuscript technically sound, and do the data support the conclusions?

**Response:** We would like to thank the reviewers, the comment is well received. The information missing from the conclusion regarding the ‘survival time’ is included in this version of the manuscript.

2. Has the statistical analysis been performed appropriately and rigorously?

**Response:** We believe that we have conducted every appropriate statistical analysis based on the type of research questions/objective of the study.i.e Kaplan–Meier is used to estimate the cumulative hazard of virological failure at different time points and Multivariate Cox regression analysis was used to assess predictors of virological failure.

3. Have the authors made all data underlying the findings in their manuscript fully available?

**Response:** Thank you for your comment, the data underlying the findings in the manuscript will be made available to the public at acceptance for publication.

4. Is the manuscript presented in an intelligible fashion and written in standard English?

**Response**: The comment is well received and the authors made every effort to improve the quality of English.

5. *Review Comments to the Author*

**Reviewer#1**

1. The result of this study was not a novel finding. Many studies from Ethiopia and in extensive Africa cohort studies explored the several impact factors associated with virological failure (VF), which should add in reference.

**Respons**e: Thank you for the comment. Yes the authors also reported that there are similar literature on this area and are used across the document sections including background, literature review, methods, discussion etc. However, based on the requirement of the journal we included the consecutive literature used in the sections included in this manuscript only. Additionally, the predictor variables and the incidence tend to vary across settings and the fact that the problem is still an important public health problem made the researchers believe that there are more factors to be explored and considered during planning and implementation of tackling this problem/virological failure.

2. The methodology was not clear and could not follow by the STROBE checklist. For example: how to study size determined, how the loss to follow-up handled, how and when should be the censor, and how many numbers of completely follow up were analyzed.

**Response:** Thank you. The comment is well received and included in the manuscript. We have omitted the information from the manuscript in an attempt to limit the size of the document. However it is addressed in such a way: *“Sample size was determined using Epi Info™ Version: 7.2.1.0 StatCalc by considering 95 % confidence level, 80 % power, unexposed to exposed group ratio of one and taking the key predictor of VF (BMI <16 which gave the largest sample size among the variables) from a previous study in Woldia and Dessie hospitals and Waghimra zone. Therefore, the calculated minimum sample size is 308 and by considering a 10% non-response rate the final sample is 340”.* ***Censored:-*** *Those patients complete the follow up, transferred out or lost without developing virological failure*

3. In table 3, How to report the number of outcome events or summary measures over time

**Response:** Thank you for the comment. The aim of Table 3 is to indicate the association between ***outcome variable*** indicated by Event (patients who developed Virological failure) and censored (who never developed virological failure) and **predictor variables**. The cumulative hazard of outcome event which is virological failure over time is inidicated under subheading of ‘**The incidence of Virological failure’. It is stated as “***The cumulative hazard of VF at 12, 24, 36 and 48 months were 1.2%, 3.7%, 5.5%and 7.6% respectively*”.

4. The study duration was July 2016-Dec 2019, but the patient who last developed the event was 53 months, and VF reported at the end of 48 months that were not consistent with the duration of the study.
**Response**: Thank you for your observation and comment. It was typological error the study duration was July 2016 to December 2020. This comment is addressed in the document accordingly.

5. Statistical method was not precise which confounders were chosen to adjust for and why or exploratory multivariate survival was analyzed.

**Response**: Thank you for your comment. Cox proportional hazards model was used to identify covariates significantly associated with virological failure as it is one of the study objectives and used to control the confounding. It is a survival analysis regression model, which describes the relation between the event incidence, as expressed by the hazard function and a set of covariates. Bradburn, M. J., Clark, T. G., Love, S. B., & Altman, D. G. (2003). Survival analysis part II: multivariate data analysis--an introduction to concepts and methods. *British journal of cancer*, *89*(3), 431–436. <https://doi.org/10.1038/sj.bjc.6601119>

6. Reference 20 could not be comparable in contextualized higher incidence because that previous study was done in naive patients with poor CD4 and high viral load.

**Response**. Comment well received and addressed.

7. This manuscript is needed to clarify many points such as good adherence definition, OI/TB former history, or recent infection.

**Response**: comment well received and addressed in the document as follows: Adherence is defined as follows: Clients on ART with >95% adherence are considered to have good adherence, those with 85-94% adherence are fair and those with <85% are considered to have poor adherence (35). History of Opportunistic infection or Tuberculosis indicates whether the patientshas history of any Opportunistic infection including Tuberculosis. On the other hand recent infection is whether the person currently has TB or any Opportunistic infection.

8. In conclusion, Cotrimoxazole prophylaxes(CPT) are given or not depend on the CD4 level. CTP plus antiretroviral (ARV) drug could decrease viral load in cases of an initial high viral load. This study should examine subgroups and interactions among CPT, CD4 level, and ARV.

**Response** : Thank you for the comment. The comment is well received and an interaction was checked between the three and no interaction was detected.

9. The manuscript's title declared patient used the first-line drug, but the inclusion criteria had not inferred the antiretroviral type. The manuscript should be more clear.

**Response**: Thank you. It was not included previously with intention of managing the size of the manuscript. Now it is well addressed in the document as follows: *Inclusion and exclusion criteria: The inclusion criteria include: clients aged 15years or older, who were on treatment for at least ten months and on first line ART. Transfer in and those without viral load test were excluded from the study*

10. Some typo errors and English language in the discussion part might be made readers confused. This study should examine subgroups and interactions among CPT, CD4 level, and ARV.

**Response**: Thank you. Comment well addressed.

**Reviewer #2: PEER REVIEW**
1.0 Summary of the Research and Overall Impression

It was noted that in the document the author states that the use of CPT prophylaxis was associated with virological failure. The statement in the Results section, Paragraph 5, Factors Associated with Virological Failure, Line 6 “patients taking cotrimoxazole prophylaxis are 87% less likely to develop to VF compared to those who are on the prophylaxis” this statement was not clear. The authors should clarify to avoid confusion and ensure that this statement on results support the conclusion.

**Response**: Thank you for your comment. It is well addressed. It is typo error. It was corrected as follows: *Patients taking cotrimoxazole prophylaxis are 87% less likely to develop to VF compared to those who are* ***NOT*** *on the prophylaxis (AHR=0.13, 95% CI: 0.026- 0.68).*

2.0 Discussion of specific areas for improvement

Results- Paragraph 5 - Factors associated with virological failure- Line 6
1. “patients taking cotrimoxazole prophylaxis are 87% less likely to develop to VF compared to those who are on the prophylaxis”

**Response**: Comment well addressed as indicated above
