# Supplementary material for: Incidence, survival time and associated factors of virological failure among adult HIV/AIDS patients on first line antiretroviral therapy in St. Paul’s Hospital Millennium Medical College—A retrospective cohort study
Source: PLoS One. 2022 Oct 13;17(10):e0275204. doi: 10.1371/journal.pone.0275204 (PMC9560068; doi:10.1371/journal.pone.0275204)
Supplement: Attachment — Submitted filename: Response to reviewers.docx [file pone.0275204.s003.docx]

**Response to reviewers**

1) The study dates in the main body of the manuscript were previously updated, but have not been updated in the Abstract - please revise accordingly.

**Response**: Thank you for your feedback and your observation. Comment well received and corrected.

2) The previous reviewer requested further detail on how confounding variables were identified. This concern has not been fully addressed; please update your Methods section to outline how potential confounding variables were identified.

Response: Comment well received and addressed in the methods section.

3) Related to the above, please ensure that all variables reported in the Results section are specified in the Methods section and provide details of how each variable was measured.

**Response**: Thank you for your feedback, comment well addressed in the manuscript. *‘’The potential associated factors included age, gender, education, marital status, disclosure status, occupational status, BMI, base line drug regimen, cotrimoxazole prophylaxis, base line functional status, WHO stage, adherence to treatment, TB/HIV co-infection, opportunistic infections other than TB and CD4 cell count.’’*
