# Supplementary material for: Incidence, survival time and associated factors of virological failure among adult HIV/AIDS patients on first line antiretroviral therapy in St. Paul’s Hospital Millennium Medical College—A retrospective cohort study
Source: PLoS One. 2022 Oct 13;17(10):e0275204. doi: 10.1371/journal.pone.0275204 (PMC9560068; doi:10.1371/journal.pone.0275204)
Supplement: Attachment — Submitted filename: Response to reviewers.docx [file pone.0275204.s004.docx]

**Response to reviewers**

**Comment**: It remains unclear how confounding factors were identified; the Methods section only indicates that "Cox proportional hazards model was used to identify factors significantly associated with virological failure and to control confounding factors.". The specific methods to identify potentially confounding factors are not clear from this sentence. Please provide further detail

**Response**: Thank you for your comment. The details are addressed in the methods and limitation section too.
